# SART1 uniquely localizes to spindle poles forming a SART1 cap and promotes spindle pole assembly

**DOI:** 10.1016/j.jbc.2025.108561

**Published:** 2025-05-02

**Authors:** Hideki Yokoyama, Daniel Moreno-Andrés, Kaoru Takizawa, Zhenzhen Chu, Anja Scheufen, Tsumugi Funabashi, Jian Ma, Wolfram Antonin, Oliver J. Gruss, Yoshikazu Haramoto

**Affiliations:** 1 National Institute of Technology, Ibaraki College, Hitachinaka, Japan; 2 Institute of Biochemistry and Molecular Cell Biology, Medical School, RWTH Aachen University, Aachen, Germany; 3 ID Pharma Co. Ltd, Tsukuba, Japan; 4 Institute of Genetics, University of Bonn, Bonn, Germany; 5 National Institute of Advanced Industrial Science and Technology, Tsukuba, Japan; 6 Department of Agri-Production Sciences, College of Agriculture, Tamagawa University, Tokyo, Japan

**Keywords:** centrosomal protein, microtubule binding, PCM proteins, spindle pole formation, ran

## Abstract

The nuclear protein squamous cell carcinoma antigen recognized by T cells 1 (SART1) has been associated with pre-mRNA splicing, but SART1 RNAi knockdown results also in defects in mitotic progression, centrosome biogenesis, and chromosome cohesion. The mitotic roles of SART1 have not been characterized in detail, and it remains unclear whether SART1 functions in mitosis directly or indirectly *via* pre-mRNA splicing. Here, we identify SART1 as a direct, mitosis-specific microtubule-associated protein. SART1 downregulation in human cells leads to spindle assembly defects with reduced microtubule dynamics, end-on attachment defects, and checkpoint activation, while microtubule dynamics remain unaffected in interphase. SART1 uniquely localizes to the distal surface of mitotic centrosomes along the spindle axis, forming a previously not described structure we refer to as SART1 cap. Immunoprecipitation of SART1 consistently identifies centrosomal proteins as interaction partners. Immunostaining of these shows that SART1 downregulation does not affect centriole duplication and centrosome accumulation of **γ**-tubulin but reduces the accumulation of selective pericentriolar material (PCM) proteins such as ninein. Depletion of SART1 from frog egg extracts disrupts spindle pole assembly around sperm nuclei and DNA-coated beads. Spindles formed around DNA-coated beads do not contain centrosomes but still recruit PCM proteins for spindle pole assembly. We finally show that the N-terminus of SART1 is its microtubule-binding region and is essential for spindle assembly. Our data unravel a unique localization of SART1 and its novel function to recruit selective PCM proteins for spindle pole assembly in centrosomal and acentrosomal spindle assembly.

The squamous cell carcinoma antigen recognized by T cells 1 (SART1) gene was first identified and cloned as a carcinoma antigen recognized by cytotoxic T cells (1). The SART1 protein is expressed in most proliferating cells but overexpressed in some cancer cells, including epithelial cancers (2). Therefore, SART1 has been considered a potential diagnostic marker and therapeutic target in various cancers (3, 4). However, it is still unknown whether SART1 overexpression is a cause or a consequence of malignant cell transformation, and the physiological function of SART1 remains incompletely understood.

In cell free assays using HeLa cell nuclear extracts, SART1 is required for U4/U6.U5 tri-small nuclear ribonucleoprotein (snRNP) recruitment to the prespliceosome and thus plays a role in pre-mRNA splicing (5). Accordingly, SART1 is also called as U4/U6.U5 tri-snRNP–associated protein 1. In addition, SART1 was identified in genome-wide RNAi screens as a factor required for mitotic progression (6, 7). Specific RNAi screens identified SART1 as a critical factor for centriole biogenesis (8) and sister chromatid cohesion (9). SART1 knockout in mice is embryonically lethal (10). Taken together, this suggests that SART1 has crucial functions in mitosis and cell division with vital importance for organismic homeostasis and/or development. However, we still lack detailed knowledge about the molecular action of SART1 in mitosis.

In eukaryotic cells, proteins containing nuclear localization signals (NLSs) are actively imported into the nucleus. During interphase, they are retained in the nucleus and might have critical nuclear functions, such as DNA replication, transcription, and splicing (11). Upon nuclear envelope breakdown at the beginning of mitosis, however, nuclear proteins become accessible to cytoplasmic proteins, including microtubules (MTs), and some play cell cycle–specific, moonlighting roles in spindle assembly and function (11). Many nuclear proteins specifically function in the vicinity of mitotic chromatin. They are spatially activated *via* the GTP-bound form of the Ran GTPase (RanGTP) produced locally around chromatin (12). RanGTP binds to the heterodimeric nuclear transport receptor importin α/β and dissociates NLS-containing nuclear proteins from these importins. The liberated nuclear proteins are active and induce spindle assembly around chromatin (12). To identify such nuclear MT regulators, we have affinity-purified proteins that contain NLS sequences and bind to MTs (microtubule-associated proteins (MAPs)) (13). We obtained more than 200 proteins (14). The NLS-MAPs identified this way have proven to be an excellent resource for uncovering new mitotic regulators and revealing their functions (15, 16).

We show here that one of these factors is SART1, which has not been recognized as MAP so far. It uniquely localizes to the most far end of spindle poles and is required for mitotic progression and spindle assembly. Our data suggest that the primary function of SART1 at this place is to recruit several canonical spindle pole proteins to these sites during mitosis.

## Results

### SART1 is a *bona fide* MAP

**Figure 1:**
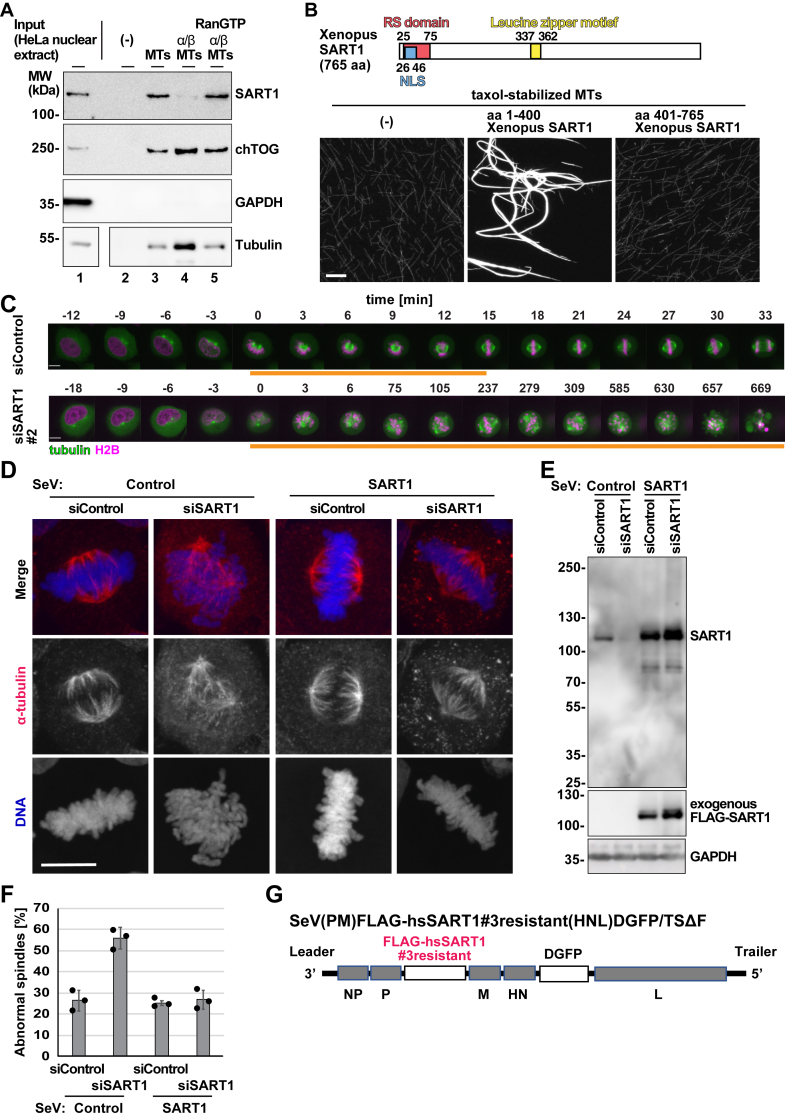
**SART1 is a MAP required for early mitotic progression and spindle assembly**. *A*, human SART1 binds to microtubules (MTs) in a RanGTP-regulated manner. HeLa nuclear extract (input) was incubated with taxol-stabilized MTs in the presence or absence of recombinant importin α/β complex and RanGTP and then centrifuged. Microtubule-associated proteins (MAPs) were eluted with high salt from the pellet. After centrifugation, and the supernatant (the eluate) was analyzed by Western blot (WB). *B*, N-terminal fragment of SART1 bundles MTs *in vitro*. Domain structure of *Xenopus laevis* SART1 contains an RS (arginine/serine-rich) domain in aa 25 to 75, a nuclear localization signal (NLS) in aa 26 to 46, and a leucine-zipper motif in aa 337 to 362. Recombinant aa 1 to 400 or aa 401 to 765 *Xenopus* SART1 was incubated with taxol-stabilized, Cy3-labeled MTs, and imaged with fluorescent microscopy. *C*, confocal images. HeLa cells expressing GFP-tubulin and mCherry-H2B were transfected with indicated siRNAs and imaged in intervals of 3 min. *Orange lines* indicate cells in prometaphase. *D*, SART1 is required for spindle assembly. HeLa cells were infected with control Sendai virus (SeV) vector or SeV carrying an siRNA#3-resistant version of human SART1. The cells were subsequently treated with control or SART1 siRNAs for 3 days, fixed, stained for α-tubulin and DNA, and imaged by a confocal microscope. Maximum-projected images are shown. *E*, Western blot of lysates from HeLa cells assayed in (*D*). *F*, quantitation of abnormal spindles assayed in (*D*). Error bars indicate SD, and *circles* indicate individual data from N = 3 experiments. n > 50 prometaphase and metaphase-like cells, based on chromatin shapes, were counted per experiment. Note that prophase, anaphase, telophase-like cells were not considered. The abnormal spindles counted may include normal prometaphase cells going to assemble spindles later. *G*, scheme of the SeV harboring FLAG-tagged human SART1, resistant to siSART1 #3, and Dasher GFP (DGFP). Scale bars represent 10 μm. SART1, squamous cell carcinoma antigen recognized by T cells 1.

We have previously identified SART1 as a potential MAP (14). To corroborate this finding and test whether SART1 can interact with MTs, we added taxol-stabilized MTs to HeLa nuclear extracts and recovered MTs and their interacting proteins by centrifugation (Fig. S1*A*). Endogenous SART1 protein in HeLa nuclear extracts was efficiently cosedimented with MTs (Fig. 1*A*, lane 3), while GAPDH was not cosedimented, indicating a specific interaction of SART1 with MTs. Addition of recombinant importin α/β complex inhibited SART1–MT interaction (lane 4). Inhibition was reversed by the co-addition of RanGTP (lane 5), which binds to importin β and prevents the importin α/β complex from binding to NLS sites. This regulation of MT binding suggests that SART1 is also a protein that regulates MTs locally around mitotic chromosomes (12). As previously reported, the MT binding of the MT polymerase chTOG, the human ortholog of *Xenopus* XMAP215, showed no inhibition by importins (16). Similarly to the situation in HeLa nuclear extracts, endogenous SART1 was cosedimented with taxol-stabilized MTs from *Xenopus* egg extracts (Fig. S1*B*, lane 4).

To examine whether SART1 directly interacts with MTs, we expressed recombinant *Xenopus* SART1 in bacteria. Because His-tagged SART1 was insoluble, an acidic-target tag (ATT) (17) was fused to SART1 to increase solubility (Fig. S1*C*). In the MT sedimentation assay (Fig. S1*A*) with purified components, HisATT-tagged SART1 bound to MTs (Fig. S1*D*), in contrast to TRIM21, which minimally bound and served as a negative control. A recombinant N-terminal SART1 fragment (aa 1–400), expressed without the ATT tag, bound to MTs in an MT sedimentation assay, while C-terminal fragments did not (Fig. S1*E*). Consistently, N-terminal, but not C-terminal, SART1 bundled MTs *in vitro* (Fig. 1*B*). These results indicated that SART1 is a *bona fide*, previously unrecognized MAP with an N-terminal MT-binding domain, which is regulated by importins and RanGTP.

### SART1 is required for mitotic progression and spindle assembly

To assess SART1 impact on mitosis, we knocked down SART1 in HeLa cells stably expressing tubulin-GFP and histone H2B-mCherry and performed live-cell imaging during mitosis. SART1 downregulation with three different siRNAs induced persistent chromosome misalignments, spindle MT assembly defects, and promethaphase-like arrest (Figs. 1*C* and S2*A*, Movies S1 and S2). Prolonged prometaphase was confirmed by CellCognition analysis (16, 18) (Fig. S2*B*). Further analysis of the live-cell data revealed that a considerable number of cells (15–45% depending on the siRNA oligo) died as a consequence of early mitotic defects (Fig. S2, *C* and *D*).

Immunostaining of the fixed siSART1-treated HeLa cells showed chromosome misalignments and spindle MT defects (Fig. 1, *D*–*F*). MTs appeared not arranged inside the spindle, and spindle poles were poorly focused. Similar spindle defects were observed with all three siRNAs (Fig. S3*A*) as well as in U2OS cells (Fig. S3*B*). In agreement with our results from live-cell imaging, we found by FACS analysis that SART1 depletion significantly induces apoptotic cell death (Fig. S3*C*). Among the three SART1 siRNAs, we further used siSART1#3 for detailed characterization of SART1, because it caused milder spindle defects (Fig. S3*A*) and the lowest numbers of cell deaths (Fig. S3*C*).

To prove the specificity of the depletion phenotype, we performed rescue experiments. We employed a Sendai virus (SeV) vector to transiently express a SART1 version resistant to downregulation by siSART1#3 oligo (19) (Fig. 1*G*). Indeed, the exogenous SART1 was well expressed in HeLa cells and fully restored the spindle defects caused by siSART1#3 treatment (Fig. 1, *D*–*F*).

### SART1 silencing reduces MT dynamics in mitosis and prevents end-on attachment of spindle MTs to chromosomes

**Figure 2:**
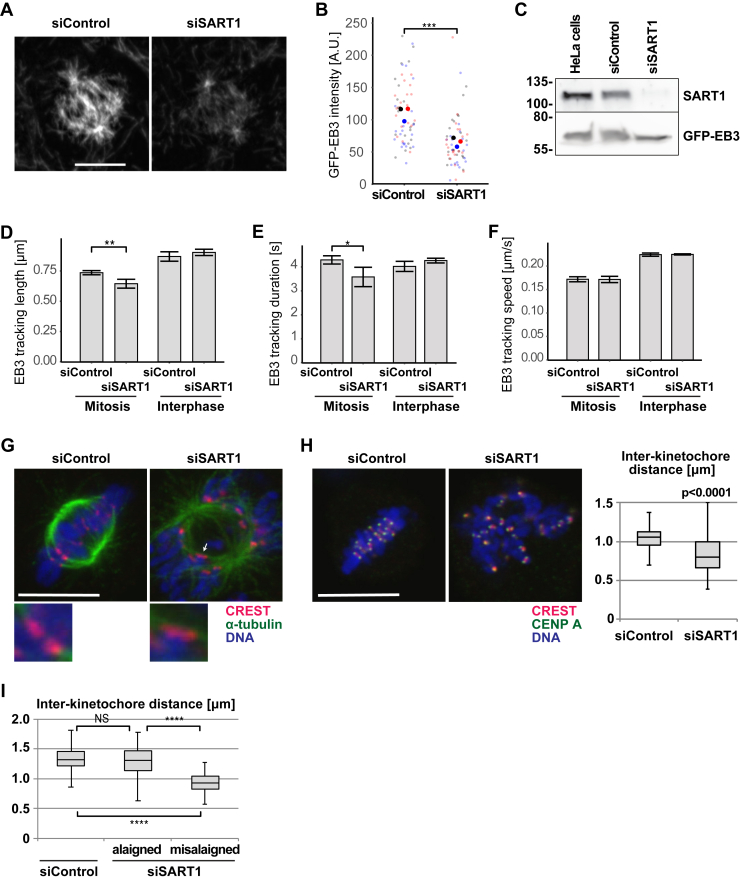
**SART1 silencing reduces microtubule dynamics in mitosis and prevents end-on attachment of spindle microtubules to chromosomes**. *A*, SART1 is required for MT plus-end binding of EB3 in mitotic MT. HeLa cells stably expressing GFP-EB3 were imaged and control cells were compared to SART1 knockdown cells. *B*, reduced EB3 plus end binding in mitotic MT in the absence of SART1. EB3 intensity as the sum of all mitotic MT (see *e*.*g*. A), was quantified with ImageJ. Overall intensity signals from 20 spindles (small dots) from three independent experiments (different colors, mean = large dots) are shown. The significance test (two-tailed students’ *t* test) compares mean values with *p* ≤ 0.001 (∗∗∗). *C*, Western blot analysis of each cell lysate using SART1 or GFP antibodies. *D-F*, MT tracking was performed in GFP-EB3 expressing control and SART1 knockdown cells at 1 s interval for 60 s and three independent experiments with quantifications of 20 to 50 individual tracks in each of 20 spindles (*i*.*e*. >500 tracks for each condition) were performed. Mean values of these experiments are shown and compared using a two-tailed Students’ *t* test with *p* ≤ 0.05 (∗) or *p* ≤ 0.01 (∗∗). *D*, MT length is reduced during mitosis in the absence of SART1. *E*, MT growing time is reduced during mitosis in the absence of SART1. *F*, MT growing speed is unchanged in the absence of SART1. *G*, end-on MT-kinetochore interactions are impaired upon SART1 downregulation. HeLa cells were treated with the siRNAs, fixed, and stained for the kinetochore marker CREST as well as α-tubulin, and DNA. Confocal slice images are shown. Note that end-on attachment of MTs to kinetochores is seen in control cells but could not upon SART1 downregulation. Three-fold magnified images of the kinetochores (indicated by an *arrow* in the case of siSART1) are shown below. *H*, interkinetochore distance is reduced upon SART1 downregulation. The RNAi-treated cells were stained for the centromere marker CENP-A, the kinetochore marker CREST, and DNA. Confocal slices were used to find kinetochore pairs to measure interkinetochore distance. n > 94 kinetochore pairs. A two-tailed Students’ *t* test reveals statistical significance with *p* ≤ 0.0001. *I*, interkinetochore distance is reduced specifically in misaligned chromosomes. CREST signals were used to measure the distance. In siSART1 cells, aligned and misaligned chromosomes are separately analyzed. n = 122 kinetochores (siSART1: 51 aligned and 71 misaligned chromosomes). Scale bars represent 10 μm. MT, microtubule; SART1, squamous cell carcinoma antigen recognized by T cells 1.

To understand the nature of the spindle defects, we tracked MT association and movement of EB3, a plus-end MT marker, by live imaging of mitotic cells (20). EB3 spindle/MT association was significantly reduced upon SART1 knockdown (Fig. 2, *A* and *B*, Movies S3 and S4). EB3 level remains constant in the knockdown cells (Fig. 2*C*), suggesting a reduction of MT polymerization events in the absence of SART1. SART1 silencing also decreased track length and duration of MT growth, while growth speed was hardly affected (Fig. 2, *D*–*F*). Importantly, the reduction of track length and duration was seen for mitotic but not interphase MTs (Fig. 2, *D*–*F*).

**Figure 3:**
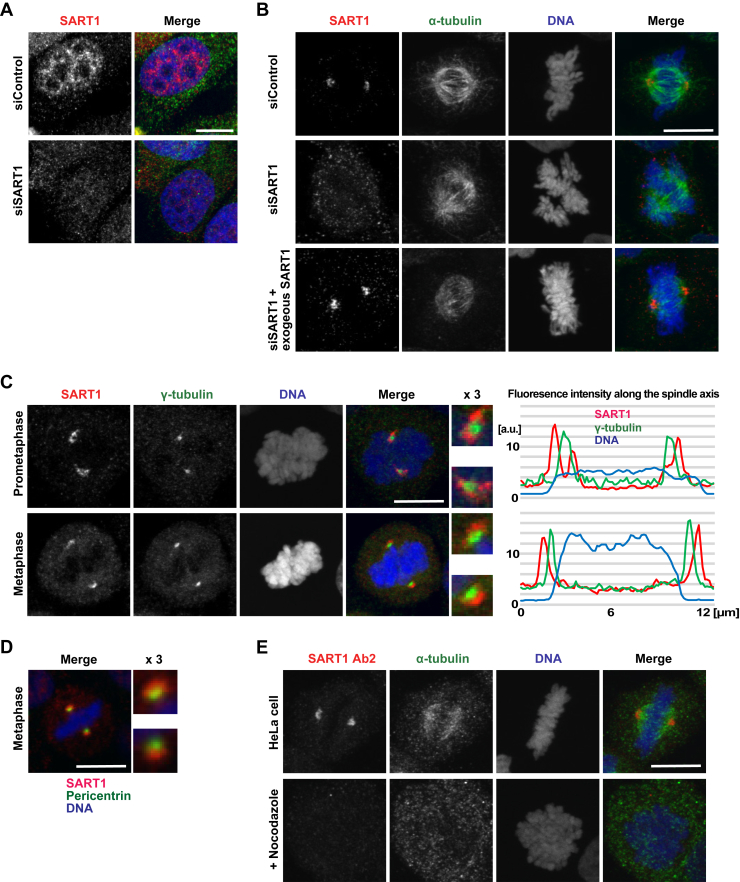
**SART1 localizes to a specific subdomain of mitotic centrosomes**. *A* and *B*, HeLa cells were treated with control or SART1 siRNAs for 3 days, fixed, and stained for SART1 (*red*), α-tubulin (*green*), and DNA (*blue*). *A*, in interphase, SART1 localizes within nucleus. *B*, SART1 localizes to spindle poles. Note that SART1 signals disappear in SART1 RNAi cells, validating the SART1 localization found in control cells. *C*, SART1 localizes around γ-tubulin in early mitosis, but eventually forms a half circle around the γ-tubulin distant from the spindle axis. HeLa cells were fixed, and stained for SART1, γ-tubulin, and DNA. Fluorescence intensity was quantified along the spindle axis using ImageJ. *D*, SART1 localizes outside of pericentrin along the spindle axis in metaphase. HeLa cells were fixed, and stained for SART1, pericentrin, and DNA. *E*, the SART1 localizes at spindle poles in the presence of MTs. HeLa cells were treated with nocodazole for 10 min before fixation and stained for SART1 α-tubulin, and DNA. Scale bars represent 10 μm. SART1, squamous cell carcinoma antigen recognized by T cells 1; MT, microtubule.

To examine the nature of chromosome misalignments, we immunostained SART1-depleted cells with human CREST autoimmune serum, which specifically labels kinetochores. In control cells, kinetochore pairs were connected to MTs emanating from both spindle poles, suggesting end-on attachment (Fig. 2*G*). By contrast, in SART1-depleted cells, majority of kinetochores appears unattached to MTs (Fig. 2*G*). Functional end-on attachments generate tension to separate kinetochores of sister chromatids, increasing the interkinetochore distance (21). Compared to control, however, interkinetochore distances did not seem to increase upon SART1 knockdown (Fig. 2*G*). To confirm this quantitatively, we costained the cells for CREST and CENP-A, an inner kinetochore marker and histone H3 variant incorporated into centromeric DNA, and measured the distance of two CENP-A dots. Whereas control cells showed an average interkinetochore distance of 1.1 μm, in SART1-depleted cells, the distance was reduced to 0.8 μm (Fig. 2*H*). Staining with the outer kinetochore marker Ndc80/Hec1 also revealed a shorter interkinetochore distance upon SART1 depletion (control 1.6 μm and depletion 1.1 μm, Fig. S3*D*). Some of the chromosomes aligned to the metaphase plate even in the absence of SART1 (such as in Fig. 3*B*). We measured the interkinetochore distance of aligned or misaligned chromosomes separately (Fig. 2*I*). We found that aligned chromosomes show comparable interkinetochore distance to control cells, but have a longer distance than misaligned chromosomes (Fig. 2*I*). Consistently, the mitotic checkpoint protein BubRI remained localized at the metaphase spindle, specifically on misaligned chromosomes (Fig. S3*E*), indicating the spindle assembly checkpoint is active in the absence of SART1. Analysis of chromosome spreads showed proper sister chromatid cohesion in our depletion condition (Fig. S3*F*). These results indicate that SART1 is not required for sister chromatid cohesion but is essential for establishing and/or maintaining MT-kinetochore attachment.

### SART1 uniquely localizes to specific surface of the centrosomes in an MT-dependent manner

To understand the precise subcellular localization of SART1 actions in mitosis, we examined the localization of SART1 in HeLa cells. A human SART1 antibody stained the nucleus and partially the cytoplasm in interphase cells as reported (22) (Fig. 3*A*), but labeled the spindle poles in mitosis (Fig. 3*B*). The staining was lost upon SART1 downregulation and restored by expressing siRNA-resistant SART1 (Fig. 3*B*) demonstrating its specificity. After nuclear envelope breakdown, SART1 localized around 2 MT asters at prophase and accumulated further around the arising spindle poles at prometaphase (Fig. S4*A*). The spindle pole localization was most prominent in metaphase, remained during anaphase, and eventually disappeared in telophase (Fig. S4*A*).

The major determinants of spindle poles in somatic cells are centrosomes that function as the primary MT-organizing center. Each centrosome consists of two centrioles surrounded by the pericentriolar material (PCM) (23). Costaining with the PCM marker γ-tubulin revealed that SART1 localized around γ-tubulin in early mitosis (prometaphase in Fig. 3*C*). But in metaphase, SART1 only covered the distal surface of the γ-tubulin along the spindle axis (Fig. 3*C*). The SART1 signal was similarly detected around pericentrin, another PCM marker (Fig. 3*D*). The same SART1-pericentrin configuration was also observed using a different SART1 antibody (Fig. S4*B*).

Since we have proved SART1 as a MAP, we examined how MTs affect the spindle pole localization of SART1. Before fixation, HeLa cells were treated for 10 min with nocodazole to depolymerize MTs. In this condition, SART1 disappeared from the spindle poles (Fig. 3*E*), indicating a requirement of MTs for the SART1 localization. This contrasts with the pole localization of γ-tubulin, which remained largely unchanged upon nocodazole treatment (Fig. S4*C*).

These results indicate that SART1 accumulates around centrosomes during early mitosis and, once the spindle establishes, localizes to the distal surface of centrosomes along the spindle axis. This localization is dependent on MTs.

### SART1 interacts with centrosomal proteins and recruits selective PCM proteins for spindle pole assembly

To understand the mechanism of how SART1 localizes to centrosomes and how SART1 functions there, we immunoprecipitated SART1 from *Xenopus* egg extracts using a specific antibody that we generated (Figs. S4*A* and S5*A*). SART1-interacting proteins were eluted from the antibody beads with a high pH buffer (Fig. S5*B*). These conditions released SART1 interaction partners from the beads, while SART1 itself remained mostly on the beads (Fig. S5*B*), presumably due to strong antigen–antibody interaction. Analysis of the eluate by shotgun mass spectrometry identified known interacting partners of SART1 involved in mRNA splicing (SF3B1, SF3B2, IK, SF3B4, RBM25, SF3B5, PRPF40A, CHERP, DDX42, ZMAT2, SNRNP70, PRPF6, SF3A3, SNRPA, DDX46; Table S1) (24). As expected, SART1 itself was found with a low mass spectrometry score. Intriguingly, mass spectrometry identified numerous centrosomal proteins (Cep192, APC, POC1, Cep44, Cep43, Cep152, Cep63, Plk1, SSX2IP, Cep97, Cep85, POC5, CDK11B, Cep70, and centrin 3; Table S1).Similar mass spectrometry data were obtained in three biological repeats.

**Figure 4:**
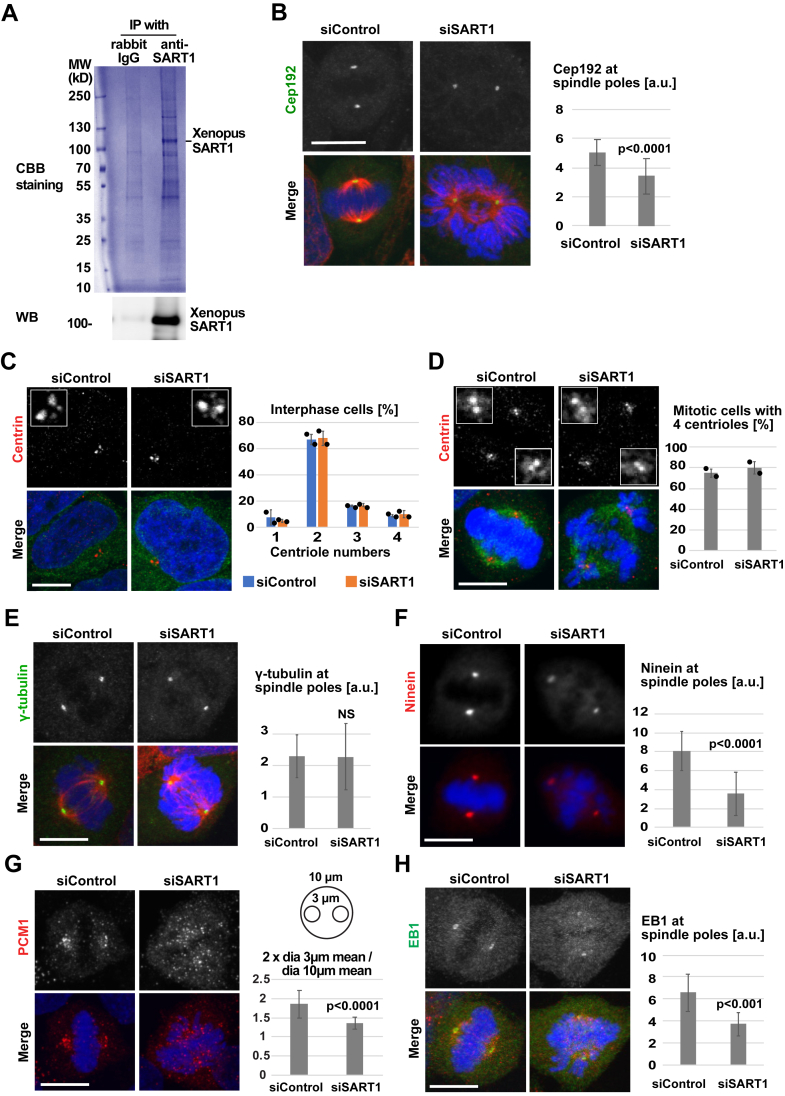
**SART1 is required for recruitment of multiple centrosomal proteins**. *A*, immunoprecipitation (IP) of SART1 from *Xenopus* egg extract using rabbit polyclonal antibodies raised against *Xenopus* SART1, analyzed by SDS-PAGE and Coomassie staining. Note the SART1 signal at 110 kDa, confirmed by Western blot. *B*, reduction of Cep192 at mitotic centrosomes in SART-downregulated HeLa cells. siRNA-treated cells are stained for Cep192 (*green*), α-tubulin (*red*), and DNA (*blue*). Cep192 intensity at spindle poles was quantified using ImageJ. N = 2 experiments, n = 10 cells per experiment. *p* value (student’s test, two tailed). *C* and *D*, centrosome duplication is not impeded by SART1 downregulation. siRNA-treated HeLa cells were stained for the centriole marker centrin (*red*), α-tubulin (*green*), and DNA (*blue*). Centriole numbers are counted per cell. Error bars indicate SD, and *circles* indicate individual data from N = 2 experiments, n = 50 cells per experiment. *C*, interphase. *D*, mitosis. *E*, no change of γ-tubulin at centrosomes. siRNA-treated HeLa cells were stained for γ-tubulin (*green*), α-tubulin (*red*), and DNA (*blue*). γ-tubulin intensity at spindle poles were quantified. N = 2 experiments, n = 10 cells per experiment. *F*, reduction of ninein at centrosomes. siRNA-treated cells were stained for ninein (*red*), and DNA (*blue*). Ninein intensity at spindle poles was quantified. N = 2 experiments, n = 10 cells per experiment. *G*, disperse of PCM1 over the mitotic cell upon SART1 downregulation. siRNA-treated cells were stained for PCM1 and DNA. Accumulation of PCM1 at spindle poles was calculated as ratio of PCM1 intensity at the spindle poles (two 3 μm circles) and a larger area around chromatin (10 μm circle). N = 2 experiments, n = 10 cells per experiment. *H*, EB1 centrosome enrichment is reduced upon SART1 depletion. siRNA-treated cells were stained for EB1 (*green*), α-tubulin (*red*), and DNA (*blue*). EB1 signal at spindle poles was quantified. N = 2 experiments, n = 10 cells per experiment. *p* values (Student’s test, two tailed). NS (not significant). Scale bars represent 10 μm. EB, end-binding; SART1, squamous cell carcinoma antigen recognized by T cells 1; PCM, pericentriolar material.

Following its identification with the highest score among the centrosomal proteins, we examined the localization of Cep192 in SART1-downregulated HeLa cells. Cep192 is an integral component of centrosomes required for both centriole duplication and centrosome maturation (25, 26). Immunostaining with a Cep192 antibody showed that Cep192 was partially (∼30%) lost from centrosomes upon SART1 knockdown (Fig. 4*B*). Because Cep192 is essential for centriole duplication (26), we wondered if SART1 depletion also affects centriole duplication. Immunostaining of the centriole marker centrin showed, however, that the number of centrioles was unaffected by SART1 depletion (Fig. 4, *C* and *D*). As in the control (27), we mostly detected two centriole dots in interphase and four dots (two for each spindle pole) in mitosis, indicating proper centrosome duplication in the absence of SART1 (Fig. 4, *C* and *D*).

It is known that the knockdown of Cep192 prevents centrosome maturation by abolishing the recruitment of PCM proteins containing the MT nucleation factor γ-tubulin (25). SART1-depleted cells did not show reduced amounts of γ-tubulin at centrosomes (Fig. 4*E*), while the localization of ninein, PCM1, and EB1 was significantly impaired (Fig. 4, *F*–*H*). EB1 is a plus-end marker of MTs, but an important centrosome component to anchor MTs (28). The reduction of EB1 at centrosomes (Fig. 4*H*) is consistent with the decreased incorporation of the related protein EB3 in the live-cell imaging (Fig. 2*A*). We wondered whether SART1 downregulation changes the amounts of PCM proteins and, therefore, performed quantitative PCRs and Western blots. The mRNA level of ninein and PCM1, and the protein level of EB1 were unchanged in the absence of SART1 (Figs. S2*A* and S5*C*). One of the two quantitative PCR primers was designed over two exons (Table S1), indicating also that mRNA splicing of ninein and PCM1 was not impaired, at least between these exons (Fig. S5*C*). In agreement with the notion that its protein level was unchanged, PCM1 was dispersed throughout the mitotic cell in the absence of SART1 (Fig. 4*G*).

These data reveal that SART1 depletion does not affect centriole numbers or γ-tubulin recruitment, but significantly reduces the localization of specific PCM components at centrosomes. It was recently shown that 50% reduction of Cep192 does not inhibit the mitotic progression in HeLa cells (29). These data altogether suggest that distinct from Cep192, SART1 is specifically required for recruitment of selective PCM proteins to mitotic centrosomes.

### SART1 promotes spindle bipolarization *via* its N-terminal MT-binding region

**Figure 5:**
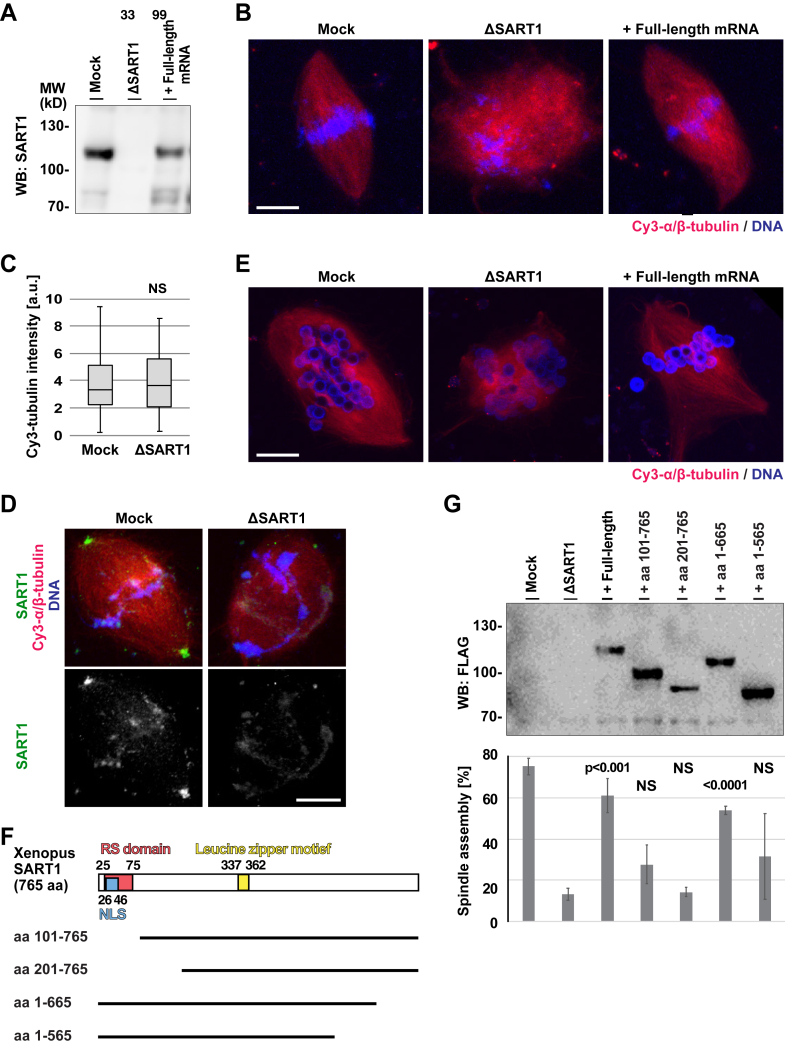
**SART1 is required for spindle pole assembly in *Xenopus* egg extracts and its N-terminus is essential for this function**. *A*, depletion and add back of SART1, examined by Western blot. *Xenopus laevis* egg extract was treated with control (mock) or SART1 antibody-coated beads. The resulting extracts were used for the cell cycle reaction in the presence or absence of SART1 mRNA. *B*, SART1 is required for spindle assembly. Mock- and SART1-depleted extract shown in (*A*) were incubated with sperm, Cy3-labeled tubulin (*red*) in the presence or absence of SART1 full-length mRNA, and cycled to interphase and mitosis. DNA was stained with DAPI. Note that not clear spindle poles were found in depletion and the bipolarity was re-established by expressing recombinant SART. *C*, SART1 depletion does not affect MT amounts assembled around chromatin. Cy3-tubulin intensity around chromatin was quantified. N = 3 experiments, n = 10 structures per experiment. *p* values (Student’s test, two tailed). NS (not significant). *D*, SART1 localizes to spindle poles in *Xenopus* egg extracts. Cycled sperm spindles were assembled as in (*B*), and stained with *Xenopus* SART1 antibody and DAPI. *E*, SART1 is essential for spindle assembly around DNA-beads. Mock- and SART1-depleted extract was incubated in DNA-coated beads and Cy3-tubulin in the presence or absence of SART1 mRNA, and cycled for spindle assembly. Note that even the spindles without centrosomes, SART1 is required for spindle pole assembly. *F*, domain structure of *Xenopus laevis* SART1 and its deletion mutants constructed. *G*, the N-terminal, NLS-containing region of SART1 is critical for bipolar spindle assembly. SART1-depleted extract was supplemented with indicated SART1 mRNAs and used for spindle assembly as shown in (*B*). Expression of each exogenous protein was confirmed by Western blot. Frequency of bipolar spindles was counted. N = 3 experiments, n > 50 DNA structures per experiment. *p* values against ΔSART1 extract (Student’s test, two tailed). NS (not significant). Scale bars represent 10 μm. MT, microtubule; SART1, squamous cell carcinoma antigen recognized by T cells 1.

We used *Xenopus* egg extracts to further understand the molecular function of SART1. In this cell-free system, spindle assembly can be faithfully recapitulated when the extracts are supplemented with sperm heads and are cycled into interphase and then to mitosis (16, 30). Our *Xenopus* SART1 antibody, used for the immunoprecipitation (Fig. 4*A*), efficiently depleted endogenous SART1 from egg extracts (Fig. 5*A*). While control antibody-treated (mock) extracts assembled bipolar spindles with chromosomes aligned at the metaphase plate, depletion of SART1 caused defects in spindle assembly (Fig. 5*B*). Although MT polymerization around sperm chromatin was quantitatively similar to the control extracts (Fig. 5*C*), spindle bipolarity was not established, and chromosomes did not align to the spindle center (Fig. 5*B*). These spindle abnormalities were rescued by addition of *Xenopus* SART1 mRNA to express recombinant SART1 (Fig. 5, *A* and *B* and *G*).

Consistent with our findings in human cells, the *Xenopus* SART1 antibody stained spindle poles and, importantly, this staining disappeared upon SART1 immunodepletion (Fig. 5*D*). Using egg extracts, spindles can also be assembled around DNA-coated beads (Fig. 5*E*) (30). These artificial spindles do not contain centrioles but still accumulate PCM proteins for spindle pole formation (31). Here, SART1 depletion resulted in spindle bipolarization defects (Fig. 5*E*), and expression of recombinant SART1 also restored bipolarity (Fig. 5*E*). This demonstrates that SART1 is crucial for spindle bipolarization also in the absence of centrioles.

**Figure 6:**
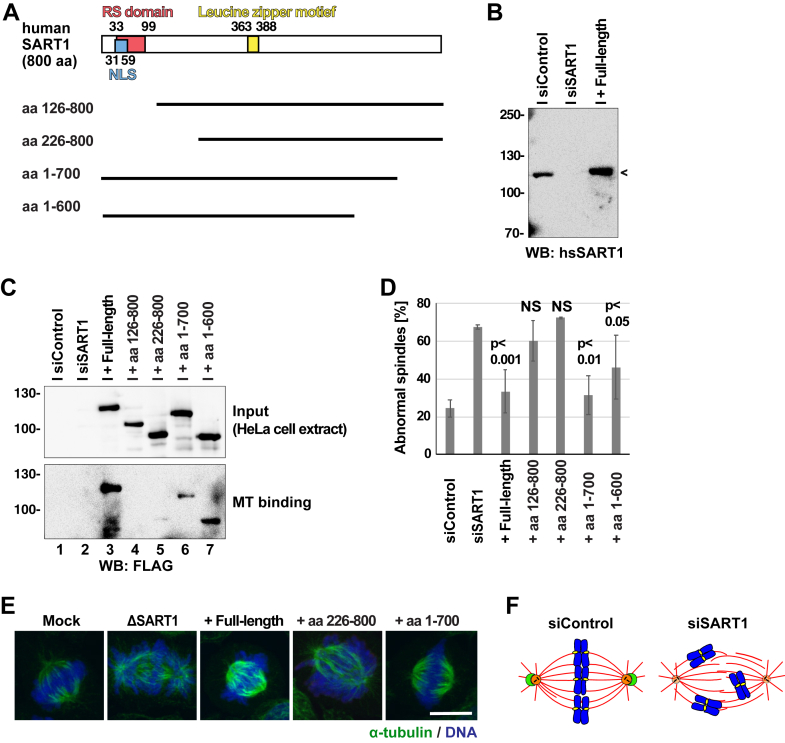
**N-terminal, MT-binding region of SART is also important for spindle assembly in human cells.***A*, domain structure of *Homo sapiens* SART1 and its deletion mutants constructed. Human SART1 contains an RS (arginine/serine-rich) domain in aa 33 to 99, a nuclear localization signal (NLS) in aa 31 to 59, and a leucine-zipper motif in aa 363 to 388. *B*, Western blot of HeLa cells transfected with control siRNA, SART1 siRNA, or SART1 siRNA + SART1 full-length construct. *C*, Western blot of HeLa cell extracts transfected with control siRNA, SART1 siRNA, or SART1 siRNA + the constructs shown in (*A*). The cell extracts (input) were incubated with taxol-stabilized MTs and centrifuged to detect MT binding. Note that full-length and fragments lacking the C-terminus bound (*C*, lane 3, 6, and 7), but fragments lacking the N-terminus did not (lane 4 and 5). *D*, quantitation of abnormal spindles assayed in (*C*), with criteria described in Figure 1*F*. Error bars: SD. N = 3 experiments, n > 50 prometaphase and metaphase-like cells per experiment. Note that among the constructs, the N-terminus–depleted SART could not restore spindle assembly. *E*, representative images of spindles assembled in (*C*). The scale bar represents 10 μm. *F*, a model explaining spindle abnormalities in SART1 knockdown cells and the physiological functions of SART1 in spindle assembly. SART1 is a is a MAP with N-terminal MT-binding region, localizes to the SART1 cap in a MT-dependent manner, and is required for spindle assembly and chromosome alignment. It remained to be addressed: SART1’s critical target protein in centrosomal maturation and whether the SART1 cap structure is important for the maturation or not. MT, microtubule; SART1, squamous cell carcinoma antigen recognized by T cells 1.

To understand which parts of SART1 are important for its function in spindle assembly, we constructed N- and C-terminal deletion mutants of the protein (Fig. 5*F*) and expressed them in SART1-depleted egg extracts (Fig. 5*G*). Besides full-length SART1, the fragment lacking the last hundred amino acids at C-terminus (aa 1–665) restored bipolar spindle assembly around sperms (Fig. 5*G*). In contrast, SART1 fragments lacking the N-terminus did not, which suggests that the N-terminal MT-binding domain (Fig. S1*D*) is important for spindle bipolarization. To gain further evidence for this hypothesis, we constructed human SART1 fragments and tested their ability to rescue the spindle abnormality in HeLa cells (Fig. 6*A*). While full-length SART1 as well as fragments lacking the C-terminus significantly restored spindle assembly, the fragments lacking the N-terminus did not restore the WT phenotype (Fig. 6, *B–E*). We could not faithfully detect the localization of SART1 fragments in the transfected cells by immunofluorescence (IF) (IF), because the FLAG antibody nonspecifically stained centrosomes even in nontransfected HeLa cells. Therefore, we prepared lysates from transfected cells and performed an MT sedimentation assay with taxol-stabilized MTs. In agreement with our hypothesis, full-length SART1 and fragments lacking the C-terminus bound to MTs (Fig. 6*C*, lanes 3, 6, and 7), but fragments lacking the N-terminus did not (lanes 4 and 5). All these results in *Xenopus* egg extracts and human cells show that the N-terminus of SART1 is a MT-binding domain critical for spindle pole assembly.

## Discussion

SART1 has been previously identified in RNAi screens as a protein required for mitotic progression (6, 7) and centrosome biogenesis (8). However, mitotic and centrosomal functions of SART1 have not been characterized in detail. It remained unclear whether SART1 is required for those events directly or indirectly *via* pre-mRNA splicing of other relevant factors. Here, we demonstrate that SART1 acts as a *bona fide* RanGTP-regulated MAP (Fig. 6*F*). The full-length HisATT-tagged SART1 partially binds to MTs, while the N-terminal His-tagged SART1 fragment show stronger binding. The ATT, introduced for better solubility of full-length SART1, is negatively charged (17) and may reduce SART1 binding to MTs, which are also negatively charged (32). SART1 localizes next to centrosomes and is required for centrosome maturation and spindle assembly. Some splicing factors (Cdc5l and the Cdc5l-containing Prp19 complex) have been shown to be similarly important for spindle assembly (33, 34). Although these proteins do not localize to specific mitotic structures, their functions on spindle assembly are independent of mRNA splicing (34). Thus, our results support the idea that multiple splicing factors have moonlighting functions on MTs in mitosis (11).

Downregulation of SART1 significantly reduces the number of MT plus-ends, MT length, and MT lifetime. Importantly, these defects are specifically detected in mitosis and not in interphase. This can be explained by the fact that SART1 resides inside the nucleus in interphase and only becomes accessible to MTs during mitosis. Ndc80, a major component of the KNM complexes, appeared to localize normally to outer kinetochores (Fig. S3*D*), suggesting proper kinetochore assembly (35). Therefore, the mitotic defects of MT plus-ends, represented by the reduction of EB3 incorporation, could be the cause of the kinetochore MT attachment defects. The attachment defects likely lead to the observed chromosome misalignment, reduced interkinetochore distance, and spindle assembly checkpoint activation (36) (Fig. 6*F*). Although amounts of EB1/EB3 are significantly reduced in spindles, overall MT mass remained unchanged after SART1 depletion in human cells and egg extracts. The results are consistent with the report that depletion of EB1 from *Xenopus* egg extracts does not affect overall MT mass but induces chromosome misalignment (37). SART1 has also been described as a pre-mRNA splicing factor required for sister chromatid cohesion (9). The reduced interkinetochore distance of unaligned chromosomes and the results of chromatin spreads show, however, that chromatid cohesion is proper in our SART1-depleted condition.

Upon nuclear envelope breakdown, SART1 localizes around the two centrosomes in prophase and further accumulates there in prometaphase. Only when bipolar spindles are established during metaphase, SART1 forms a half circle covering only the distal surface of centrosomes along the spindle axis (Fig. 6*F*). This unique SART1 localization is dependent on the presence of MTs, suggesting that SART1 is not an intrinsic component of centrosomes such as γ-tubulin. We are not aware of another protein with a similar localization pattern as SART1 at the centrosomal periphery. Therefore, we propose to name this novel centrosomal structure as SART1 cap, which is potentially important for centrosomal maturation and spindle assembly. Considering this cap-like structure is only established at metaphase, however, SART1 may function for spindle assembly initially around chromosomes through the activation by RanGTP (12). In any case, how the SART1 cap forms is an interesting question for future investigations. We envision that pulling forces move SART1 toward the cell cortex and establish the structure, similar to how outer kinetochores are stretched to opposite directions upon end-on MT attachment (21, 38).

We consistently found by immunoprecipitation that SART1 interacts with a number of splicing factors and centrosomal proteins (Cep192, APC, POC1, Cep44, Cep43, Cep152, Cep63, Plk1, SSX2IP, Cep97, Cep85, POC5, CDK11B, Cep70, and centrin 3) in *Xenopus* egg extracts. Those splicing factors have not been reported to localize on spindle poles. We found that SART1 depletion reduces the accumulation of selective centrosomal proteins at centrosomes (Fig. 6*F*), including Cep192, ninein, PCM1, and EB1, while it did not affect the recruitment of the major MT nucleation factor γ-tubulin. Although Cep192 depletion is known to abolish both centriole duplication and centrosome maturation (25, 26), SART1 depletion, in which Cep192 was ∼30% reduced, did not affect centriole duplication and the amounts of γ-tubulin at centrosomes. It has been shown that 50% reduction of Cep192 does not inhibit the mitotic progression in HeLa cells (29). These results suggest that the reduction of the PCM proteins (ninein, PCM1, and EB1) in SART1-depleted cells is not caused by the partial reduction of Cep192, but due to a direct effect of the SART1 absence. PCM proteins recruited by SART1 could promote MT dynamics and, as a result, contribute to chromosome alignment (36, 39).

While spindles are severely defective in SART1-depleted cells, the amount of spindle MTs seem unaffected. Indeed, in SART1-depleted egg extracts, we find similar spindle defects as in human cells with MT amounts not statistically different from control spindles. This phenotype is consistent with the fact that γ-tubulin accumulation at centrosomes is not affected in SART1-depleted human cells. Thus, SART1 is not required for MT nucleation, but essential for spindle bipolarization by recruiting selective PCM proteins (Fig. 6*F*). The pole assembly defects are also detected in egg extract spindles assembled around DNA-coated beads. Importantly, spindles assembled around the DNA beads do not contain centrosomes, but still recruit PCM proteins for the spindle pole assembly (31). Taken together, we conclude that SART1 is not required for centrosome *de novo* formation and MT assembly, but essential for centrosome maturation and spindle bipolarization.

Both in egg extracts and human cells, full-length SART1 (*Xenopus* or human, respectively) rescues spindle assembly defects caused by SART1 depletion, while SART1 fragments lacking the N-terminus do not. Consistently, while full-length SART1 expressed in human cells binds to MTs in a sedimentation assay, the N-terminus–deleted fragments do not. Furthermore, the N-terminal fragment of *Xenopus* SART1 binds directly to MTs and bundles preexisting MTs *in vitro*. MT bundling is usually caused by dimerization of a MAP, as represented by PRC1 and Tau (40, 41). SART1, therefore, could form dimers. It should be also noted that RNase A treatment did not inhibit the N-terminus–mediated MT bundling *in vitro*, suggesting that RNA is not involved in the MT bundling induced by SART1. Several nuclear proteins bind MTs *via* their NLS and neighboring residues and regulate MT functions with the other protein domains (32). Indeed, the N-terminus of SART1 contains a predicted NLS (aa 26–46 of *Xenopus* SART1, aa 31–59 of human SART1 (42). Therefore, we conclude that the N-terminus of SART1 contains the MT-binding region and is essential for spindle pole formation (Fig. 6*F*). Human SART1 has an RS (arginine/serine-rich) domain, shared by the SR protein family of splicing factors, in aa 33 to 99 (43). It also contains a leucine-zipper motif in aa 363 to 388 (43). However, it has not been reported which domain/motif of SART1 is required for RNA splicing, and it will be interesting to address it in the future.

In summary, our results unravel SART1 as a novel RanGTP-regulated MAP that localizes to the outer surface of centrosomes along the spindle axis, which we designate as SART1 cap. SART1 promotes the recruitment of specific centrosomal components for spindle pole assembly that is important for MT dynamics and proper chromosome alignment.

## Experimental procedures

### Recombinant proteins and antibodies

A cDNA covering the full-length *Xenopus laevis* SART1 (NM_001086027.1) was *in vitro* synthesized (GenScript) and subcloned into pET28a (Novagen) with NdeI and XhoI sites. For antibody production, the recombinant *Xenopus* SART1 protein was expressed in BL21 (DE3) *Escherichia coli* and solubilized from inclusion bodies with 6 M urea. The protein was purified with Ni-NTA Agarose (Qiagen), dialyzed to PBS containing 6 M urea, and used for immunization in rabbits (Hokkaido System Science). From the antisera, *Xenopus* SART1 antibody was purified using the antigen column and used for Western blot and IF at 1 μg/ml. To increase recombinant *Xenopus* SART1 solubility, HisATT, modified from Flag-ATT (17), was synthesized (Genscript) and subcloned into the pET28a-xlSART1 plasmid *via* the NcoI and NdeI sites. The HisATT-SART1 was expressed in BL21 (DE3) and purified with Ni-NTA, and dialyzed to CSF-XB buffer (10 mM K-Hepes, 100 mM KCl, 3 mM MgCl_2_, 0.1 mM CaCl_2_, 50 mM sucrose, and 5 mM EGTA, pH 7.7) containing 10% glycerol and 1 mM DTT, and used for MT sedimentation assay. *Xenopus* SART1 fragments were amplified by PCR, cloned into pET28a, and prepared as HisATT-SART1. For *in vitro* translation in egg extracts, *Xenopus* SART1 and its depletion mutants were amplified by PCR and subcloned into a pCS2+ vector harboring the FLAG tag sequence at the N-terminus. Importin α, importin β, and RanQ69L-GTP were expressed in *E*. *coli* and purified with TALON beads (15).

The following published and commercial antibodies were used: rabbit (Rb) anti-chTOG for Western blot (WB) at 1 μg/ml, Rb anti-α-tubulin (15) for IF at 2 μg/ml, anti-γ-tubulin (20) (Rb, IF 1 μg/ml), ninein serum (20) (Rb, IF 1:500), PCM1 (20) (Rb, IF 1:1000), XCAP-G (16) (Rb, WB 1 μg/ml), α-tubulin (mouse (Ms) B-5-1-2; Sigma, IF 1:2000), BubR1 (Ms, BD, IF 1:500), CENP-A (Ms, Enzo, IF 3 μg/ml), centrin (Ms, Millipore, IF 1:500), Cep192 (Rb, Bethyl, IF 1 μg/ml), CREST (human, Antibody Inc., IF 1:200), EB1 (Ms, BD, IF 1:250), FLAG (Ms M2, Sigma, WB 1:1000), γ-tubulin (Ms GTU-88, Sigma, WB 1:1000, IF 1:500), GAPDH (Ms, Santa Cruz, WB 1:2000), Ndc80 (Ms, GeneTex, IF 1:500), phospho-Histone H3 (Rb, Millipore, IF 1:500), SART1 #1 (Ms, Abcam, IF 3 μg/ml, WB 1 μg/ml), and SART1 #2 (Ms, Santa Cruz, IF 3 μg/ml), and pericentrin (Rb, Abcam, IF 1 μg/ml). Secondary antibodies for IF were anti-rabbit IgG conjugated with Alexa Fluor 488 or 568, and anti-mouse IgG conjugated with Alexa Fluor 488 or 568 (above from Life technologies, 1:1000), and anti-human IgG conjugated with CF568 (Biotum, 1:1000). DNA was counterstained with DAPI at 1 μg/ml.

### SeVs and plasmid vectors for rescue experiments in human cells

Human SART1 mutant resistant to siSART1#3 (s228453) was designed by introducing the following silent mutations into the ORF of SART1 cDNA (NM_005146.5): A813G; T819C; T822C; C825T; and C828T. The mutant cDNA with N-terminal FLAG tag was synthesized *in vitro* (Genewiz) and cloned into pSeV/TSΔF (44) (Fig. S2*E*). To reconstruct virus, the plasmid DNA was transfected to LLC-MK2 cells stably expressing F (fusion) protein.

The Human SART1 mutant resistant to siSART1#3 and its deletions mutants (amplified by PCR) were subcloned into pCI-neo Mammalian Expression Vector (Promega).

### MT sedimentation and bundling assays

HeLa nuclear extract (4C Biotech) was diluted to 1 mg/ml with CSF-XB buffer, and centrifuged at 20,000×*g* for 10 min at 4 °C. The supernatant was incubated with 2 μM pure taxol-stabilized MTs at room temperature (RT) in the presence or absence of recombinant 3 μM importin α/β complex and 5 μM RanQ69L-GTP, a dominant positive mutant of Ran locked in the GTP-bound state, and pelleted at 20,000 g for 10 min 20 °C. MAPs were eluted from the pellet with CSF-XB supplemented with 500 mM NaCl for 5 min. After another centrifugation, the supernatant (eluate) was analyzed by Western blot.

*Xenopus* CSF egg extracts were diluted 1:3 with CSF-XB buffer. After centrifugation at 20,000×*g* for 10 min at 4 °C, the supernatant was incubated at RT in the presence or absence of 4 μM taxol-stabilized MTs for 15 min. The samples were centrifuged at 20,000×*g* for 10 min at 20 °C, and pellets were incubated with CSF-XB supplemented with 500 mM NaCl for 5 min, and centrifuged again. The resulting supernatant (eluate) and pellet were analyzed by Western blot.

0.1 μM recombinant SART1 was incubated with 2 μM taxol-stabilized MTs for 15 min at RT and centrifuged at 20,000×*g* for 10 min at 20 °C. The Supernatant and pellet were analyzed by Coomassie staining or Western blot. The assay was also done in the presence or absence of recombinant 3 μM importin α, 3 μM importin β, and 5 μM RanQ69L-GTP.

To examine MT bundling activity, 0.3 μM Cy3-labeled MTs was incubated with ∼1 μM recombinant *Xenopus* SART1 aa 1 to 400 in 10 μl BRB80 (80 mM Pipes, 1 mM MgCl_2_, 1 mM EGTA, pH 6.8) buffer for 10 min at RT. The samples were squashed between slides and coverslips with fixative. Images were acquired by using an Olympus Fluoview FV1000 confocal microscope.

To examine the MT binding ability of SART1 fragments expressed in HeLa cells, cells were lysed in radioimmunoprecipitation assay buffer (50 mM Tris pH 8.0, 150 mM NaCl, 50 mM sodium orthovanadate, 1% v/v NP40, 0.1 mM PMSF) supplemented with complete protease inhibitor cocktail (Roche). The cell extract (200 μg) was incubated with 2 μM taxol-stabilized MTs for 15 min at RT and centrifuged at 20,000×*g* for 10 min at 20 °C. The pellet containing MTs and MAPs was analyzed by Western blot.

### Cell culture, transfection, IF, and microscope

HeLa and U2OS cell lines were obtained from the American Type Culture Collection and cultured in Dulbecco's modified Eagle's medium supplemented with 2 mM GlutaMAX, 10% fetal bovine serum, and 500 units/ml penicillin-streptomycin (all from Gibco). The knockdown experiments were performed with the following siRNA oligonucleotides: siSART1#1 (s17343), 5′-GGCUCAACAUGAAGCAGAAtt-3′, siSART1#2 (s17345), 5′-CCCAAUACAGCUUACCGUAtt-3′, and siSART1#3 (s228453). AllStars siRNA (from Qiagen) was used as negative control. HeLa and U2OS cells were grown on 12 mm round coverslips (Marienfeld) and transfected with 10 nM of each siRNA using Lipofectamine RNAiMAX (Invitrogen) according to the manufacturer's instructions. After 72 h, cells were fixed with Mildform 10 N (Wako) at RT for 15 min and stored at 4 °C. For immunostaining with BubRI and centrin antibodies, cells were fixed with cold methanol, instead of Mildform, at −20 °C for 10 min. Unless otherwise stated, siSART1#3 was used for the functional analyses of SART1, including the rescue experiments.

For rescue experiments, HeLa cells were infected with SeVs at multiplicity of infection 3. After 8 h incubation, medium was replaced with fresh one containing 20 nM siRNAs, and cells were cultured for additional 72 h. Alternatively, HeLa cells were transfected with pCI-neo-hsSART1 full-length and deletion mutants with 1 ng/μl using ViaFect Transfection Reagent (Promega). After 4 h incubation, the siRNA and Lipofectamine mixture was added to the cells and incubated for additional 72 h.

When indicated, HeLa cells were treated with 20 μM nocodazole for 10 min and fixed with Mildform.

For IF staining, fixed cells were incubated with blocking buffer (PBS + 2% bovine serum albumin + 0.1% Triton-X100) at RT for 30 min and then incubated with the primary antibodies in the blocking buffer at 4 °C overnight. Cells were washed with PBS, incubated with the secondary antibodies and DAPI at RT for 30 min, washed again with PBS, and mounted with Mowiol 4 to 88 (Calbiochem). To quantify abnormal spindles objectively, we considered prometaphase cells, which may be going to progress to metaphase, as abnormal cells. Thus, the abnormal spindles contain normal prometaphase cells.

Fluorescence images were acquired by using an Olympus Fluoview FV1000 confocal microscope equipped with a UPlanSApo 60x/1.35 Oil objective at 0.5 μm Z steps. Maximum intensity projections were obtained using ImageJ (NIH; https://imagej.net/ij/). Confocal slices are used to detect kinetochore MT attachment and sister kinetochore pairs. Interkinetochore distance was measured using ImageJ. Maximum projected images are used to quantify fluorescent intensity using ImageJ. Accumulation of PCM1 at spindles poles was calculated as ratio of PCM1 signal intensity at poles (two 3 μm circles) and larger area around chromatin (10 μm circle) (Fig. 4).

### Live-cell imaging experiments

HeLa cells expressing H2B-mCherry (18), a gift from Daniel Gerlich (IMBA) were transfected with 20 μM siRNA oligonucleotides in 8-well μ-slide chambers (Ibidi) and, after 30 h, were imaged for 48 h in a Axioobserver Z1 (Zeiss) equipped with a heating and CO_2_ incubation system (Ibidi), Colibri LED illumination (Zeiss), a CCD camera (AxioCamMR3; Zeiss), and a Plan-Apochromat 10 × NA 0.45 M27 objective. Single position files were converted into image sequences with the AxioVision software (LE64; V4.9.1.0; Zeiss; https://www.micro-shop.zeiss.com/en/us/system/software-axiovision+software-products/1007/). Afterward, segmentation, annotation, classification of cells was performed using the CellCognition software (http://www.cellcognition.org/software/cecoganalyzer) (16, 18). More than 100 cell mitotic trajectories per condition were used for quantification and analysis with Microsoft Excel (https://www.microsoft.com/ja-jp/microsoft-365/excel) and GraphPad Prism (https://www.graphpad.com/features).

HeLa cells expressing GFP-tubulin and H2B-mCherry (18), also from Daniel Gerlich, were transfected with 20 μM siRNA oligonucleotides in 8-well μ-slide chambers (Ibidi) and, after 30 h, were imaged with a Ti2 Eclipse microscope (Nikon) equipped with an X-light spinning disk, a LED light engine SpectraX (Lumencor), GFP/mCherry filter sets and a Plan-Apochromat 40 × NA 0.95 objective. A software-based autofocus module from the AR-Elements software (Nikon; https://www.microscope.healthcare.nikon.com/products/software/nis-elements/nis-elements-confocal) was used to perform confocal fluorescence imaging of the single best-in-focus optical section of cells undergoing mitosis every 3 min. Image galleries from mitotic cell trajectories were assembled using FiJi (https://imagej.net/software/fiji/) and mounted for figures using Inkscape (Free Software Foundation, Inc).

To image MT plus ends, HeLa EGFP-EB3–expressing cells (45), a gift from Jan Ellenberg, were seeded into ibidi 8-well chambers (ibidi GmbH). Imaging was performed on a Zeiss LSM 880 confocal microscope with a 63/1.4 NA oil-immersion objective in a homemade 37 °C microscope incubator using medium without Phenol Red (Thermo). Time-lapse images were acquired with a 600-ms exposure at a temporal resolution of 1000 ms for 60 s. Cropping of single frames was performed within ImageJ using a Gaussian blur filter of radius two and analyzed with a multiple particle tracking software (45, 46). After processing raw data with ImageJ, we used a MATLAB-based program to detect and track the tips of polymerizing MTs. Tracking algorithms are as described previously (45, 46).

### Flow cytometry

Three days after siRNA transfection, cells were stripped with trypsin and stained with a FITC Annexin V Apoptosis Detection kit (BD) following manufacture’s instruction, and analyzed using a CytoFLEX flow cytometer (Beckman Coulter) and FlowJo (FlowJo, LLC; https://www.flowjo.com/).

### Chromosome spreads

HeLa cells were transfected with the indicated siRNAs and grown for 72 h. To enrich mitotic cells, 330 nM nocodazole was added and incubated for 4 h. Cells were stripped by trypsin, washed with PBS, incubated with 75 mM KCl at RT for 8 min. An equal volume of Carnoy’s fixative (methanol: acetic acid = 3:1) was added and centrifuged to remove the supernatant. The Carnoy wash was repeated two more times. Cells were resuspended in Caynol, spotted on heated slide glass drop by drop, and stained with DAPI for microscopy. Images were taken using an Olympus Fluoview FV1000 confocal microscope.

### Quantitative real-time PCR

Three days after siRNA transfection, total RNAs were isolated using PureLink RNA Mini Kit (Ambion) and were subjected to PureLink DNase (Invitrogen) treatment. Reverse transcription and quantitative real-time PCR were conducted using TaqMan RNA-to-Ct one-step Kit and 7500 Fast Real-Time PCR System (both from Applied Biosystems) with the indicated primer and probe sets (Table S2). To amplify only spliced mRNAs, one of the primers or probes was designed on exon-intron junction. Values were normalized to those of GAPDH.

### *Xenopus* egg extracts and cell-free assay

Cytostatic factor–arrested M-phase *X*. *laevis* egg extracts (CSF extracts) were prepared as described (30). In short, *Xenopus* eggs were dejellinated by cysteine treatment, washed with CSF-XB buffer (10 mM K-Hepes, 100 mM KCl, 3 mM MgCl_2_, 0.1 mM CaCl_2_, 50 mM sucrose, and 5 mM EGTA, pH 7.7), and crushed by centrifugation at 20,000*g* for 20 min in a SW55 Ti rotor (Beckman) at 16 °C. The straw-colored middle layer was recovered as a CSF extract. Endogenous SART1 was depleted from CSF extracts by two rounds of incubation with 60% (vol/vol) Dynabeads protein A (Invitrogen) coupled with *Xenopus* SART1 antibodies.

For spindle assembly in cycled extract (30), CSF extract was supplemented with demembraned sperm or DNA-coated beads, and incubated with Cy3-labeled tubulin, and 0.4 mM CaCl_2_ at 20 °C for 90 min to allow cell cycle progression into interphase. Samples were cycled to mitosis by addition a fresh CSF extract and incubation at 20 °C for 80 min. The spindle structures were spun down to coverslips, fixed with cold methanol, stained with DAPI in PBS, and mounted as described (30). For rescue experiments, mRNAs encoding *Xenopus* SART1 full-length and deletion mutants were prepared from the pCS2+ vectors using mMESSAGE mMachine SP6 kit (Invitrogen). The mRNAs were added to the depleted extract at the beginning of the cell cycle reactions.

MT density around sperm was quantified using ImageJ. To detect the localization of SART1, the fixed spindles on coverslips were stained with the *Xenopus* SART1 antibody and subsequently with anti-rabbit IgG conjugated with Alexa Fluor 488 and DAPI.

### Immunoprecipitation of SART1 and mass spectrometry

Dynabeads protein A was coupled with rabbit IgG or the *Xenopus* SART1 antibody following the manufacturer’s instruction. The antibody beads were cross-linked with dimethyl pimelimidate. Each bead sample (600 μl slurry) was incubated with *Xenopus* CSF egg extracts (1000 μl) at 4 °C for 60 min, washed twice with CSF-XB and twice with CSF-XB containing 0.5 M KCl and 0.1% Triton X-100. The immunoprecipitates were resuspended in SDS sample buffer and resolved by SDS-PAGE for Coomassie stain or immunoblot.

For shotgun mass spectrometry, the immunoprecipitates (600 μl bead slurry) were eluted from the beads with 0.1 M triethylamine (pH 11.5, 60 μl) and neutralized by addition of final 0.1 M Tris pH 6.8. The proteins were trypsin digested and analyzed with a Q-Exactive mass spectrometer (Thermo Fisher Scientific) at the CoMIT in Osaka University.

Tandem mass spectra were extracted for database searching. Charge state deconvolution and deisotoping were not performed. All MS/MS samples were analyzed using Mascot (Matrix Science; version 2.5.1). Mascot was set up to search the Ani_Uni_*Xenopus*_200130 database (unknown version, 57,926 entries) assuming the digestion enzyme trypsin. Mascot was searched with a fragment ion mass tolerance of 0.020 Da and a parent ion tolerance of 10.0 PPM. O + 18 of pyrrolysine and carbamidomethyl of cysteine were specified in Mascot as fixed modifications. Deamidated of asparagine and glutamine and oxidation of methionine were specified in Mascot as variable modifications.

### Statistical analyses

For live-cell imaging, data were tested for normality by D'Agostino and Pearson omnibus test. For normal distributions, ANOVA test and subsequent Dunnett multiple comparisons test were applied. When normal distributions could not be assumed, statistical significance at alpha = 0.001 was determined using a Kruskal–Wallis test followed by Dunn’s multiple comparisons test.

For other fixed cell and egg extract experiments, Student's *t* test was performed using Microsoft Excel with two-tail distribution and two-sample equal variance.

## Data availability

All data described in the article are contained within the article. In cases when mean values or representative experiments are reported, data for all individual experiments can be provided by the corresponding author upon reasonable request. Correspondence and material requests should be addressed to H. Y.

## Supporting information

This article contains supporting information.

## Conflict of interest

The authors declare that they have no conflicts of interest with the contents of this article.

## Supporting information

Supporting Informaion
