# Supplementary figures and images for: SART1 uniquely localizes to spindle poles forming a SART1 cap and promotes spindle pole assembly
Source: J Biol Chem. 2025 May 2;301(6):108561. doi: 10.1016/j.jbc.2025.108561 (PMC12155601; doi:10.1016/j.jbc.2025.108561)

Figure S1

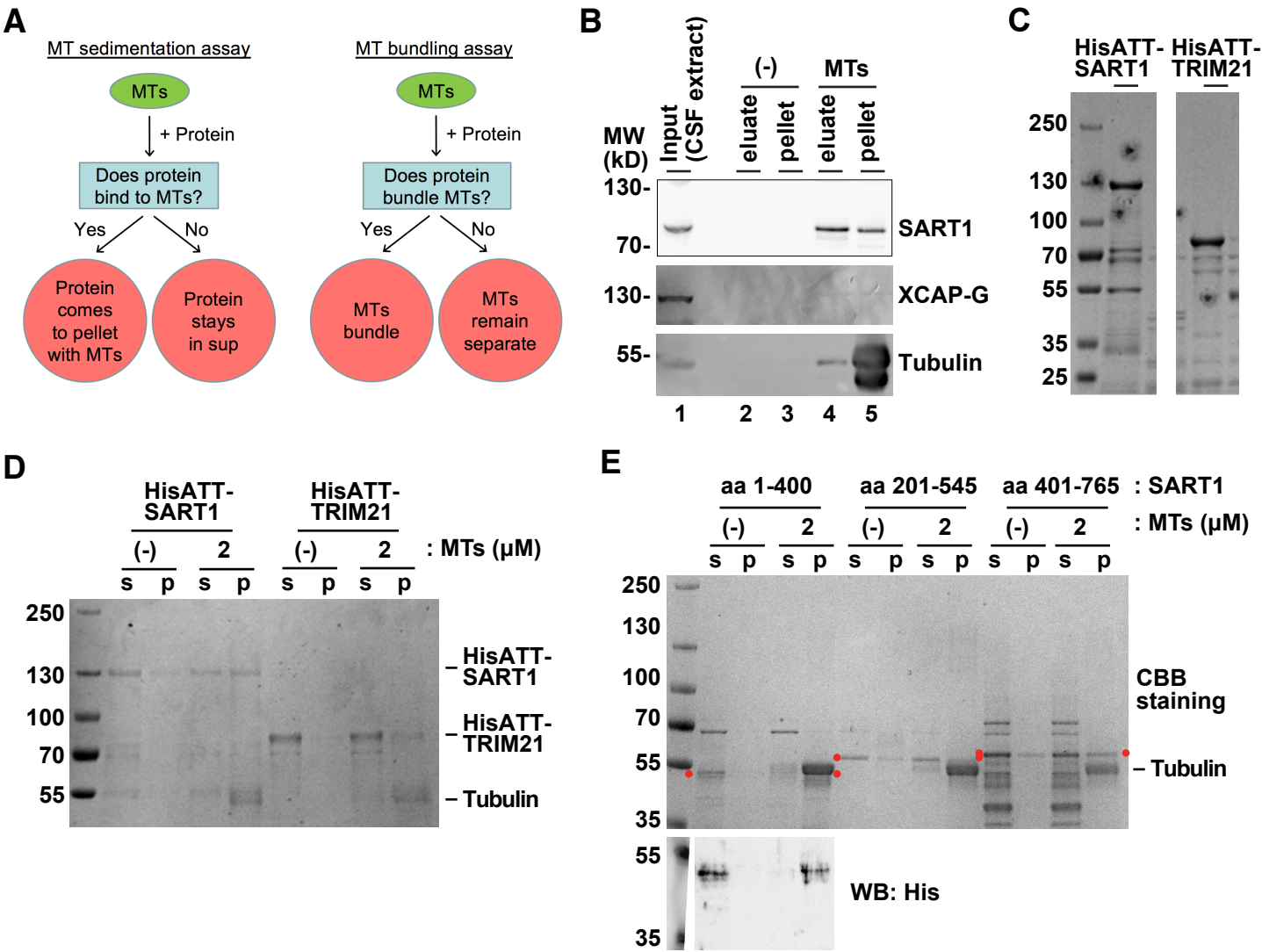

**Figure S2**

**A**

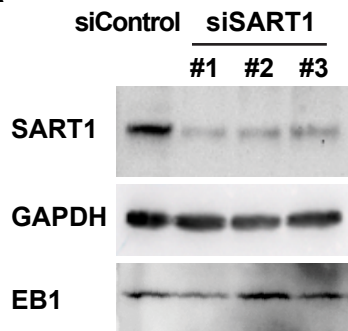

**B**

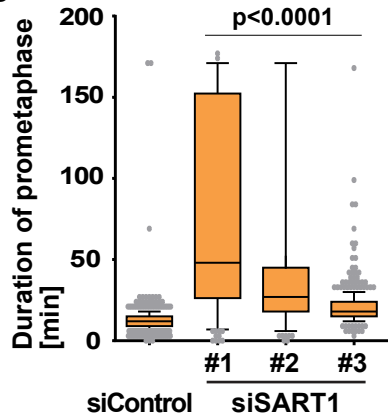

**D**

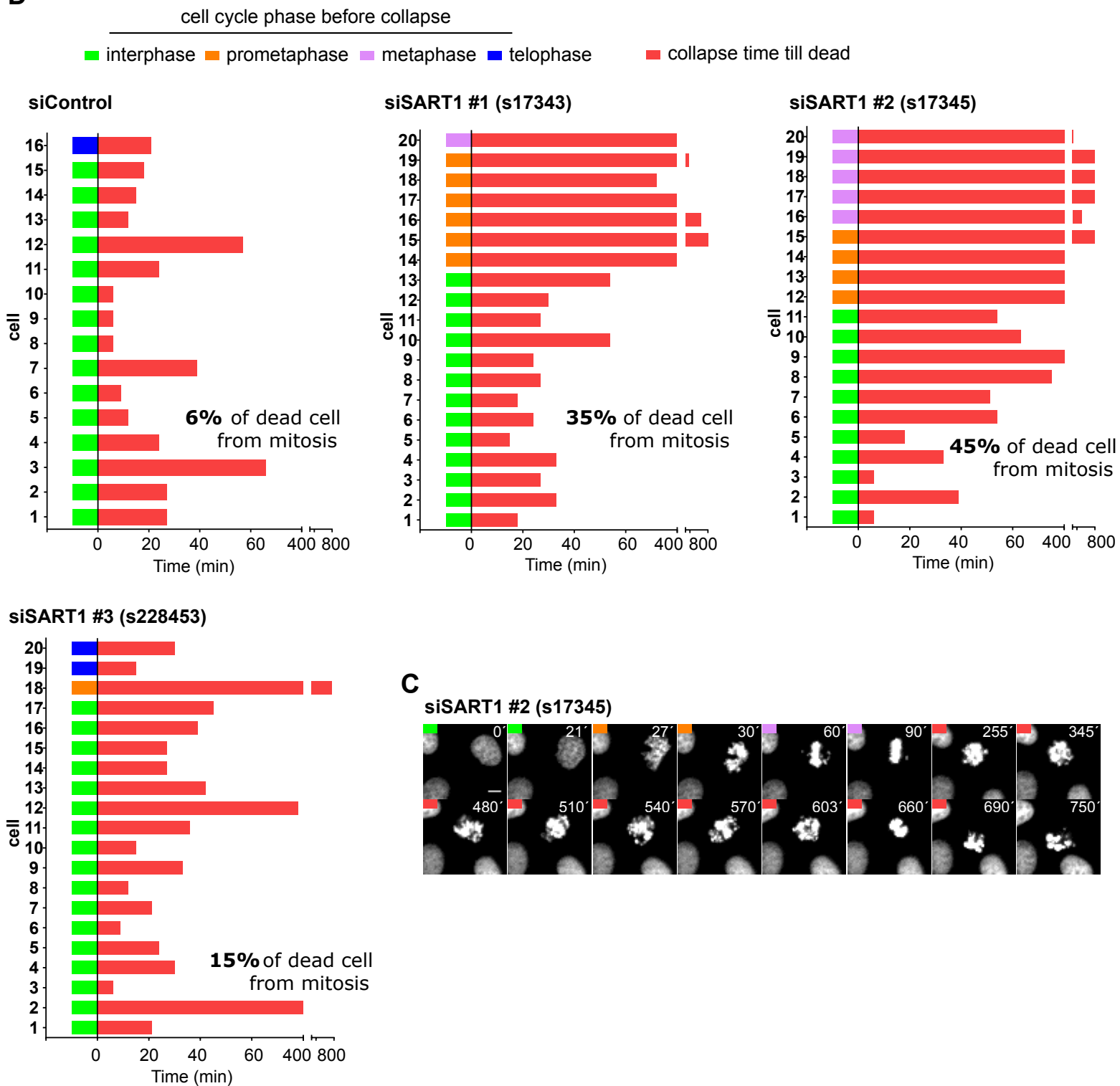

**Figure S3**

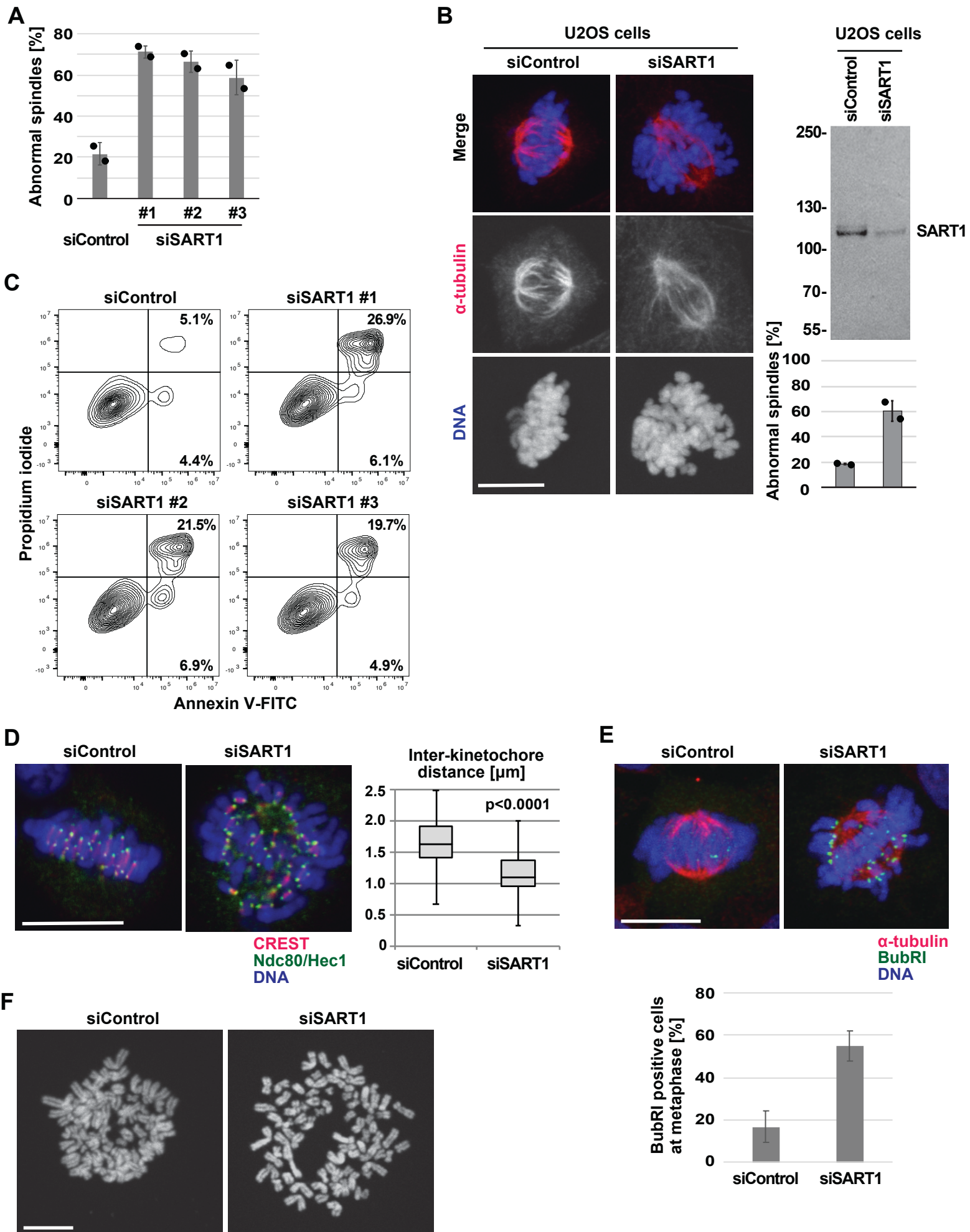

Figure S4

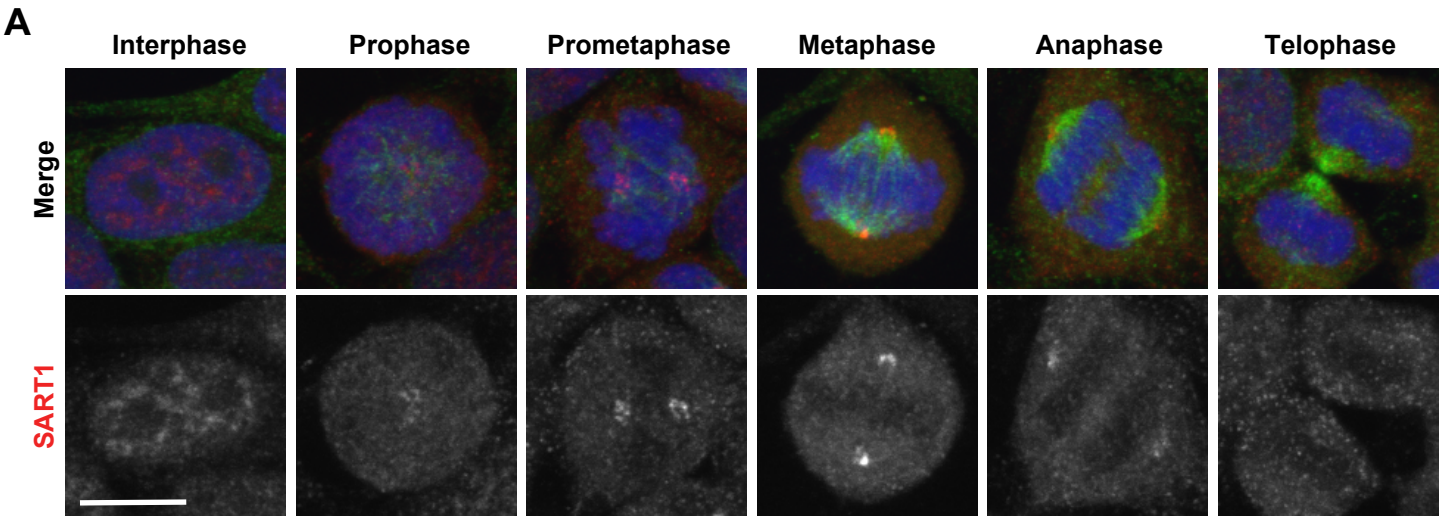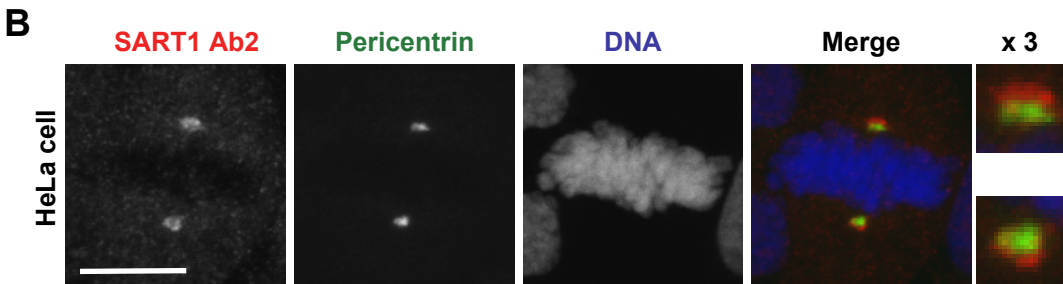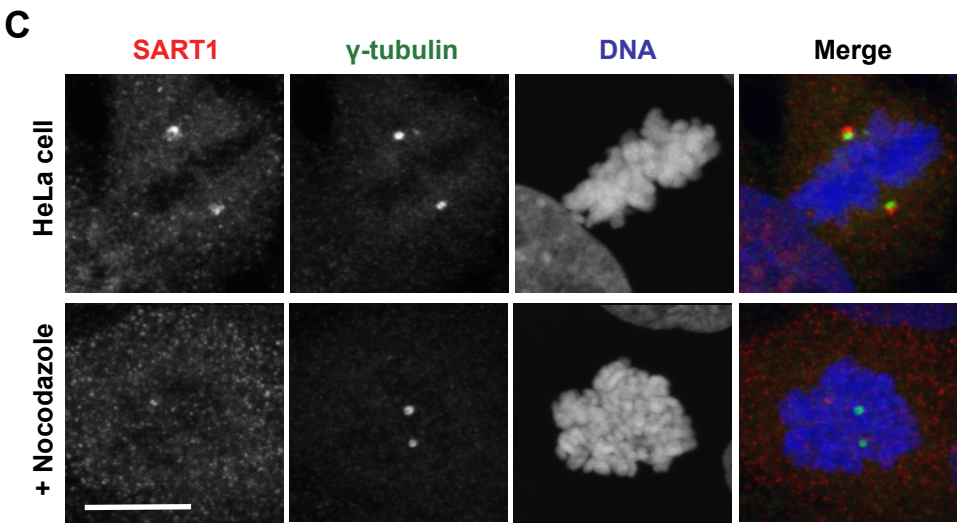

**Figure S5**

**A**

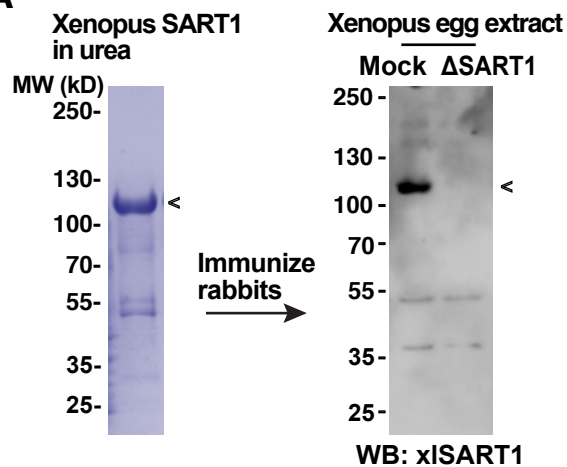

**B**

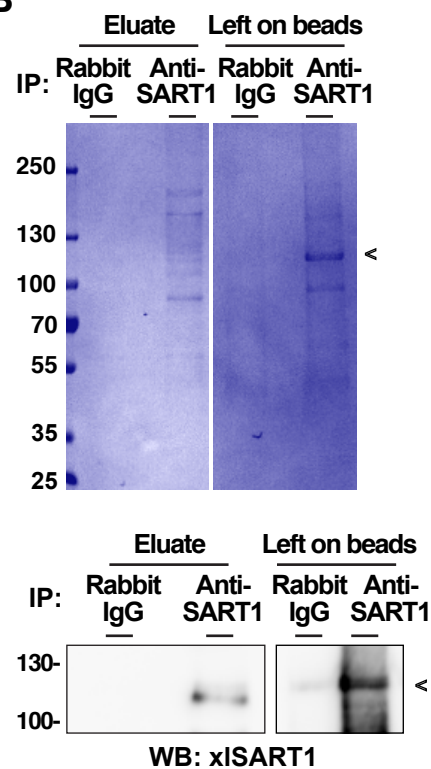

**C**

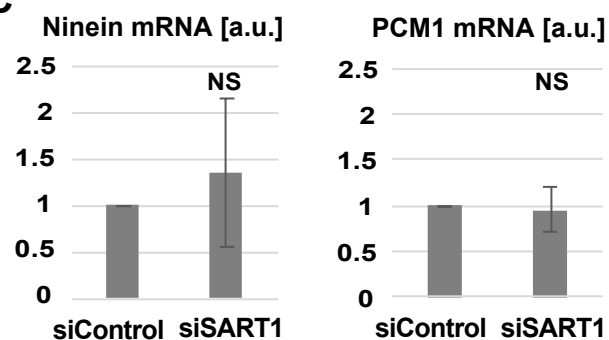

Supplement: Supplemental Figures [file mmc7.pdf]
